# Increased μ-Calpain Activity in Blasts of Common B-Precursor Childhood Acute Lymphoblastic Leukemia Correlates with Their Lower Susceptibility to Apoptosis

**DOI:** 10.1371/journal.pone.0136615

**Published:** 2015-08-28

**Authors:** Anna Mikosik, Izabella Henc, Katarzyna Ruckemann-Dziurdzińska, Joanna E. Frąckowiak, Anna Płoszyńska, Anna Balcerska, Ewa Bryl, Jacek M. Witkowski

**Affiliations:** 1 Department of Pathophysiology, Medical University of Gdańsk, Gdańsk, Poland; 2 Department of Pathology and Experimental Rheumatology, Medical University of Gdańsk, Gdańsk, Poland; 3 Clinic of Pediatrics, Hematology and Oncology, Medical University of Gdańsk, Gdańsk, Poland; INSERM-Université Paris-Sud, FRANCE

## Abstract

Childhood acute lymphoblastic leukemia (ALL) blasts are characterized by inhibited apoptosis promoting fast disease progress. It is known that in chronic lymphocytic and acute myeloid leukemias the reduced apoptosis is strongly related with the activity of calpain-calpastatin system (CCS) composed of cytoplasmic proteases—calpains—performing the modulatory proteolysis of key proteins involved in cell proliferation and apoptosis, and of their endogenous inhibitor—calpastatin. Here, the CCS protein abundance and activity was for the first time studied in childhood ALL blasts and in control bone marrow CD19^+^ B cells by semi-quantitative flow cytometry and western blotting of calpastatin fragments resulting from endogenous calpain activity. Significantly higher μ-calpain (*CAPN1*) gene transcription, protein amounts and activity (but not those of m-calpain), with calpastatin amount and transcription of its gene (*CAST*) greatly varying were observed in CD19^+^ ALL blasts compared to control cells. Significant inverse relation between the amount/activity of calpain and spontaneous apoptosis was noted. Patients older than 10 years (considered at higher risk) displayed increased amounts and activities of blast calpain. Finally, treatment of blasts with the tripeptide calpain inhibitors II and IV significantly and in dose-dependent fashion increased the percentage of blasts entering apoptosis. Together, these findings make the CCS a potential new predictive tool and therapeutic target in childhood ALL.

## Introduction

Acute lymphoblastic leukemia (ALL) is the most common type of pediatric leukemia, accounting for over 70% of all cancer cases diagnosed in patients under 15 years of age[1,2]. In addition to an enhanced cell proliferation in ALL, leukemic blasts are also characterized by reduced susceptibility to programmed cell death (apoptosis), which results in an inhibition of normal hematopoiesis and leads to the development of the disease[3–5].

Intensive development of chemotherapy together with improvements of the supportive care have contributed to a significant increase in the survival rates, which now reach more than 80% in ALL patients[2,3,6–9]. Still there are cases of refractory ALL and there are patients who relapse. Thus it remains important to search for new prognostic factors or markers of disease progression with the potential for diagnostic purposes.

We have shown before[10] that significantly reduced ability of chronic B-cell leukemia (B-CLL) cells to enter spontaneous apoptosis is associated with grossly increased total amount and activity of the proteolytic enzyme, μ-calpain, member of the so-called calpain-calpastatin system (CCS) consisting of a group of proteases–calpains—and their endogenous inhibitor–calpastatin^47^. Calpains are the cytoplasmic cysteine proteases, activation of which requires sufficiently high concentration of Ca^2+^ [11,12]. Calpain activity was first described and isolated from the rat brain in 1964[13]; it has since become the object of an intense research in the context of its (patho)physiological significance.

The main part of the CCS are so called ‘ubiquitous’ calpains: μ-calpain (calpain I) and m-calpain (calpain II), named after the concentrations of Ca^2+^ required for their full proteolytic activity in vitro: μ-calpain requires 1–100 μM Ca^2+^ and m-calpain 0.1–1 mM Ca^2+^ respectively [11,12,14]. The two-subunit structure of these classical calpains is highly evolutionarily conserved among all vertebrates, indicating the paramount importance of these proteins for the cellular function [11,14,15].

Over a hundred of important proteins have been already identified as intracellular calpain substrates [11,12,14,16–18]. Although calpains can completely hydrolyze some of their substrates, for the majority of them they do not operate like protein-degrading proteases, but modulate their activity and function by limited hydrolysis [11,12,19,20]. This adjustable biomodulation of substrates can, on one hand, lead to the transmission of signals participating in the cell cycle and cell proliferation control, but on the other hand, their role in the regulation of apoptosis (indirectly or through a direct effect on its substrates, including caspases or Bcl-2 protein family members, such as Bcl-2, Bcl-xL, Bid, Bax) is postulated [11,16,21–23].

Due to the role of calpains in abovementioned cell functions, their intracellular activation and inhibition must be precisely regulated. Uncontrolled proteolysis of calpain substrates is prevented by the only known, specific endogenous calpain inhibitor–calpastatin, exerting no influence on other enzymes, which is itself a calpain substrate [12,18,24,25]. Calpastatin is first activated through limited, Ca^2+^-dependent proteolysis by calpain. Only after such activation does calpastatin gain the ability to competitively inhibit calpain[19,21]; thus, the system operates in a feedback loop. The balance must be strictly regulated here, since the excess of one of the components of the system can lead to cell functional pathology and progression of resulting disease[21,26]. Calpains themselves can also be their own substrates in a self-limiting activation/degradation cycle[20,21].

Calpains were already shown to play a regulatory role in the formation and development of cancers, as most of their substrates are involved in cell proliferation control. Abnormal activity of calpains affects the migration and proliferation of cancer cells, as well as intra-tumour angiogenesis and apoptosis [21,27]. Engagement of calpains in human pathologies is complex as some pathologies result from the increased rate of cell death through apoptosis and some arise due to halted apoptosis and accumulation of tumor cells via biomodulation of key proteins [10,11,16,22,25,28]. Overexpression of calpains leading to a significant degradation of substrate proteins (including effector caspases) and reduced apoptosis (at least in some malignancies nullified by calpain inhibition) has been observed in several hematological disorders and derived cell lines, including the chronic lymphocytic and acute myeloid leukemias [10,29,30]. Childhood ALL blasts are also known to exhibit reduced apoptosis[31,32]. Up to date however, there are no reports on calpain system activities in these malignant cells.

Based on the facts summarized above, in this work we investigated the role of calpains in the pathomechanism of apoptosis avoidance of ALL blasts.

## Materials and Methods

### Ethics statement

The study was approved by the Local Independent Committee for Ethics in Scientific Research at the Medical University of Gdansk. The patients’ parents (legal guardians) gave written informed consent for participation in the study. The procedures were in accord with the *Helsinki Declaration of 1975*, as revised in 2008.

### Subjects

Altogether, thirty nine ALL patients diagnosed in the Department of Pediatrics, Hematology, Oncology and Endocrinology, Medical University of Gdansk, were enrolled into this study. However, due to paucity of biological material available (BM samples remaining after diagnostic BM aspiration), not all experiments could be performed on all samples. The numbers of samples included in any specific experiment are given in the figure legends. The only inclusion criteria were the diagnosis of common acute lymphoblastic leukemia and material availability. There were 19 girls and 20 boys, median age 5.68 years (from 2.5 months to 17.6 years); with no significant age differences between the genders. The bone marrows were routinely assessed by the hematological diagnostic laboratory and the leukemic blasts were classified according to their morphology and immunophenotype. All patients were diagnosed as common ALL and received treatment according to the same protocol. Detailed clinical characteristics of the patient source cohort including the karyotype analysis and treatment protocol is given elsewhere[33].

Twenty-one children in the control group were age- and gender-matched patients of the same Department, in whom the diagnostic bone marrow aspiration was performed and the malignancy was ruled out. In choice of the control group, apart from the obvious availability matter, we considered the reports indicating that B lineage progenitor (CD19^+^CD34^+^) cells form a relatively substantial proportion of the B lineage pool in the bone marrow of children younger than 15 years[34]. The only inclusion criteria were the availability of the post-diagnostic bone marrow surplus aspirate and no malignancy diagnosed. There were 10 girls and 11 boys, median age 6.5 years (from 3.5 to 17.9 years). The bone marrows were routinely assessed by the hematological diagnostic laboratory and diagnosed as: thrombocytopenia (6 patients), leukopenia (3), cyclic neutropenia (3), spherocytosis (2), deficiency anemia (2) hemolytic anemia (2), mononucleosis (1), cytomegaly (1), and collagenosis (1). In the available literature there are no reports on significant deviation of the contents and/or activity of calpains in the BM of children suffering from any of the above mentioned diseases from these seen in healthy BM.

The event-free survival was assessed two years after the initial ex vivo analysis–an event was defined as relapse (6/39 patients); of these 6 patients, one-third obtained bone marrow transplant (3/39 patients) and one-third died within the follow-up period (3/39 patients).

### Sample preparation

Bone marrow samples (BMs) from ALL patients and malignancy-free controls were obtained by aspiration biopsy; preservative-free heparin was used as an anticoagulant. All biopsies were performed prior to treatment implementation. All of the samples were processed the same way as described below, within one hour from acquisition. Bone marrow mononuclear cells (BMMC) were isolated by density gradient centrifugation on Histopaque 1077 (Sigma–Aldrich). Interphase cells were collected and washed twice in RPMI medium (Sigma–Aldrich). Aliquots of 0.3 × 10^6^ cells were used for further staining.

### Immunophenotyping

Staining to identify malignant B cells by simultaneous expression of CD19 and CD34 was chosen based on the routine bone marrow immunophenotyping performed for diagnostic purposes. The percentages of blasts in all the BMs from ALL patients were 90–100%, hence there was no need for further purification of cells for the molecular tests.

### CCS staining

All three anti-CCS antibodies (anti-μ-calpain, anti-m-calpain and anti-calpastatin) were mouse monoclonal non-conjugated antibodies (Abcam, UK, clone numbers 15C10, 107–82 and CSL5-10 respectively); rat monoclonal antibody (Becton Dickinson, USA, clone number A85-1) against mouse immunoglobulins, coupled with R-Phycoerythrin was used for the detection of these CCS antibodies in a two-step protocol. The intracellular labeling of CCS proteins was performed after staining the cells with FITC-anti-CD19/PE-Cy5-anti-CD34 mix (both from Becton Dickinson, USA, clones HIB19 I 581 respectively), fixation and permeabilisation with 2% paraformaldehyde and 0.25% saponin (Sigma Aldrich,USA) in PBS[35]. Matched isotype controls were used throughtout (Becton Dickinson, USA).

### Apoptosis assessment

Spontaneous apoptosis in ALL blasts, as well as in non-malignant BM CD19^+^ cells was measured by PE-Annexin V binding to the cell surface with simultaneous 7-aminoactinomycin D (7-AAD) staining to exclude necrotic cells (protocol by the manufacturer, Becton Dickinson, USA). Measurement of the mitochondrial membrane potential loss characteristic for earlier stages of apoptosis was performed with the JC1 probe (5,5’,6,6’-tetrachloro-1,1’,3,3’ tetraethylbenzimidazolylcarbocyanine iodide, Molecular Probes) in ALL blasts ex vivo and in 24-hours cell cultures in vitro, according to[36–38]. Separate samples of ALL blasts and non-malignant CD19^+^ lymphocytes were treated with 5 μg/ml chelerythrine (a pan-kinase C inhibitor with potent pro-apoptotic and anti-tumor activity[39]) prior to assessment of their apoptosis by JC1 staining. These experiments uniformly yielded around 98% of cells with strongly depolarized mitochondria (apoptotic) and thus served as positive control for the test.

There are reports (including ours) suggesting that human B cell lineage might contain both μ- and m-calpain[10]. Expression of both proteases in childhood ALL is put to test in this work. Currently (November 2014) a PUBMED search for “calpain AND acute lymphoblastic leukemia” or “calpain inhibition AND acute lymphoblastic leukemia” returns precisely one paper[29] describing the pro-apoptotic effect of calpain inhibitor II on established human ALL cell lines. In this work, in order to assess the effectiveness of calpain inhibition in induction of apoptosis of the ALL blasts, samples of the BMMC (2 × 10^6^ cells/ml) were cultured for 24 hours with/ or without calpain inhibitor IV (CI IV, a synthetic tripeptide aldehyde LLY: Z-Leu-Leu-Tyr-CH2F (Z = benzyloxycarbonyl), Calbiochem, UK) at concentrations ranging from 1μM to 8μM [40] or with 20 μg/mL calpain inhibitor II (CI II, N-Acetyl-Leu-Leu-Met-al; Calbiochem)[10] and tested for apoptosis induction.

The cultures were performed at 37°C in the RPMI medium supplemented with 10% FBS, 100 U/ml penicillin and 100 μg/ml streptomycin, in a 95% air, 5% CO2 humidified incubator. At the completion of incubation period, the cells were harvested, washed with PBS, stained for CD19, and processed in accordance with the JC1 staining procedure described above.

### Flow cytometry analysis

All measurements requiring flow cytometry, i.e., phenotyping and analysis of expression of the CCS member proteins, were performed using the FACScan cytometer (Becton Dickinson, USA). At least 2x10^4^ cells were acquired from each sample using the CellQuest software of the instrument and then analyzed using the Cyflogic v. 1.2.1 software. Mean fluorescence intensity (MFI) minus MFI of relevant isotype control was used as a semi-quantitative measure of antigen expression. In order to assure lack of influence of the possible FACS MFI readout instability, the instrument was calibrated at each session with the calibration beads (CaliBRITE 3 for three-color flow cytometer setup, Becton Dickinson), so as both the beads’ MFI and CV were the same throughout the project.

### Quantitative real time-PCR estimation of CCS genes’ expression

Total RNA was isolated from flash-frozen cells of ALL patients and the control group (samples @ 2 x10^6^ cells) using the miRNeasy Mini Kit (Qiagen, Netherlands) and DNA digestion was carried out using the RNase-Free DNase Set (Qiagen, Netherlands). The quality of the obtained total RNA (RNA Integrity Number) was measured by the Agilent 2100 Bioanalyzer instrument using the Agilent RNA 6000 Nano Kit and the resulting values indicated high RNA quality (>8). The reverse transcription reaction was performed using the ImProm-II Reverse Transcription System (Promega, USA). The resulting cDNA served as a template for Real-Time PCR reactions performed with the use of the LightCycler FastStart DNA Master SYBR Green I (Roche) and run on the LightCycler 2.0 instrument supplied with the LightCycler Software 4.05 (Roche Diagnostics, Germany). The sequence of primers (BLIRT, Poland) employed in the Real-Time PCR reactions were as follows: 5’-ATTTCGTTTGCTGCCTGGTG-3’ and 5’-ATGGTCAGCTGCAACCACTTA-3’ for μ-calpain, 5’-GCATACGCCAAGATCAACGG-3’ and 5’-GGAGGGGGCTTCTTCAACTC-3’ for m-calpain, 5’-CCCAAGCCTCGGAGTGAATC-3’ and 5’-AGCGGCCTTAGATTCTTCTGT -3’ for calpastatin, 5’-CAGTCAGCCGCATCTTCTTT-3’ and 5’-GACCAAATCCGTTGACTCCG-3’ for GAPDH as the reference gene. The reaction consisted of a pre-incubation step (95°C, 10 minutes), a quantification step which included 40 cycles (95°C, 10 seconds; 62°C, 10 seconds; 72°C, 5 seconds), a melting step (65°C, 15 seconds) and a cooling step (40°C, 30 seconds). The obtained Ct values of μ-calpain, m-calpain, calpastatin and the reference gene GAPDH[41] were analyzed using the ΔΔCt method and expressed as fold change over the expression of GAPDH.

### Measurement of the endogenous μ-calpain activity ex vivo

The method applied here has been described elswhere; it is based on the detection of 20–50 kDa products of specific calpastatin proteolysis by calpains[35]. The method implies that in the cells in which the activity of calpains was present in vivo, the amount of native calpastatin would be reduced, and that its degradation products would appear and be detectable in the overall amount proportional to the proteolytic activity. We have shown before that such an activity is absent from resting PBMC and that specific inhibition of calpain activity in the sample protects calpastatin from degradation[35]. Briefly, cells were lysed in EDTA-free Complete Lysis-M buffer (Roche) containing the protease inhibitor cocktail (leupeptin, aprotinin, iodoacetamide and PMSF—all at 10 μg/ml) according to the manufacturer's protocol and standard quantities of total lysate proteins were separated by SDS-PAGE. In order to demonstrate that the cleavage of calpastatin was effected by endogenous calpains in the ALL blasts, we have incubated the parallel samples of the blasts with 4 μM (IC50) calpain inhibitor IV for 24 hours prior to lysis. After electrotransfer (TRANS-BLOT Semi Dry, BioRad, USA) to a nitrocellulose membrane (PROTRAN NITROCELLULOSE, 0.45μm, Schleicher&Schuell, Germany), nonspecific binding was blocked in 3% solution of skimmed milk powder in TBS (Tris-buffered saline) with 0.05% Tween-20 for 1 hour, then twin membranes were carefully washed and incubated with either 1:1000 anti-calpastatin mouse monoclonal antibody or 1:2000 anti-actin mouse monoclonal antibody (both Abcam, Great Britain) overnight, at 4°C. Then, washed membranes were incubated with 1:2000 HRP-conjugated rabbit polyclonal antibody against mouse IgG for 2 hours. Bound antibodies were detected by chemiluminescence (SuperSignal West Pico Chemiluminescent Substrate, Thermo Scientific, USA) recorded on the x-ray film. The developed and fixed films were digitalized using the GDS-8000 System and dedicated acquisition software Labworks Image Acquisition and Analysis Software Version 4.0 (UVP Bioimaging System, UK). Relative ex vivo calpain activity was assessed by the detection and densitometric quantification of the protein bands containing the degraded fragments and native calpastatin. Densitometric analysis was performed using the Scion Image Beta (version 4.0.2) program.

### Statistical analysis

Statistical analyses were performed with STATISTICA 8 (StatSoft, Poland) software using the non-parametric Mann-Whitney U-test to compare two independent groups (relative to the groups or to the variable) after confirming the lack of normality of data distribution. All values are shown in the graphs as ‘box and whisker’ plots corresponding to median, 25^th^ and 75^th^ quartiles and data range.

## Results

### 1. Comparison of the proportions of ALL blasts and non-malignant CD19^+^ B cells containing detectable μ-, m-calpain and calpastatin and of amounts of these proteins in these cells

**Fig 1:**
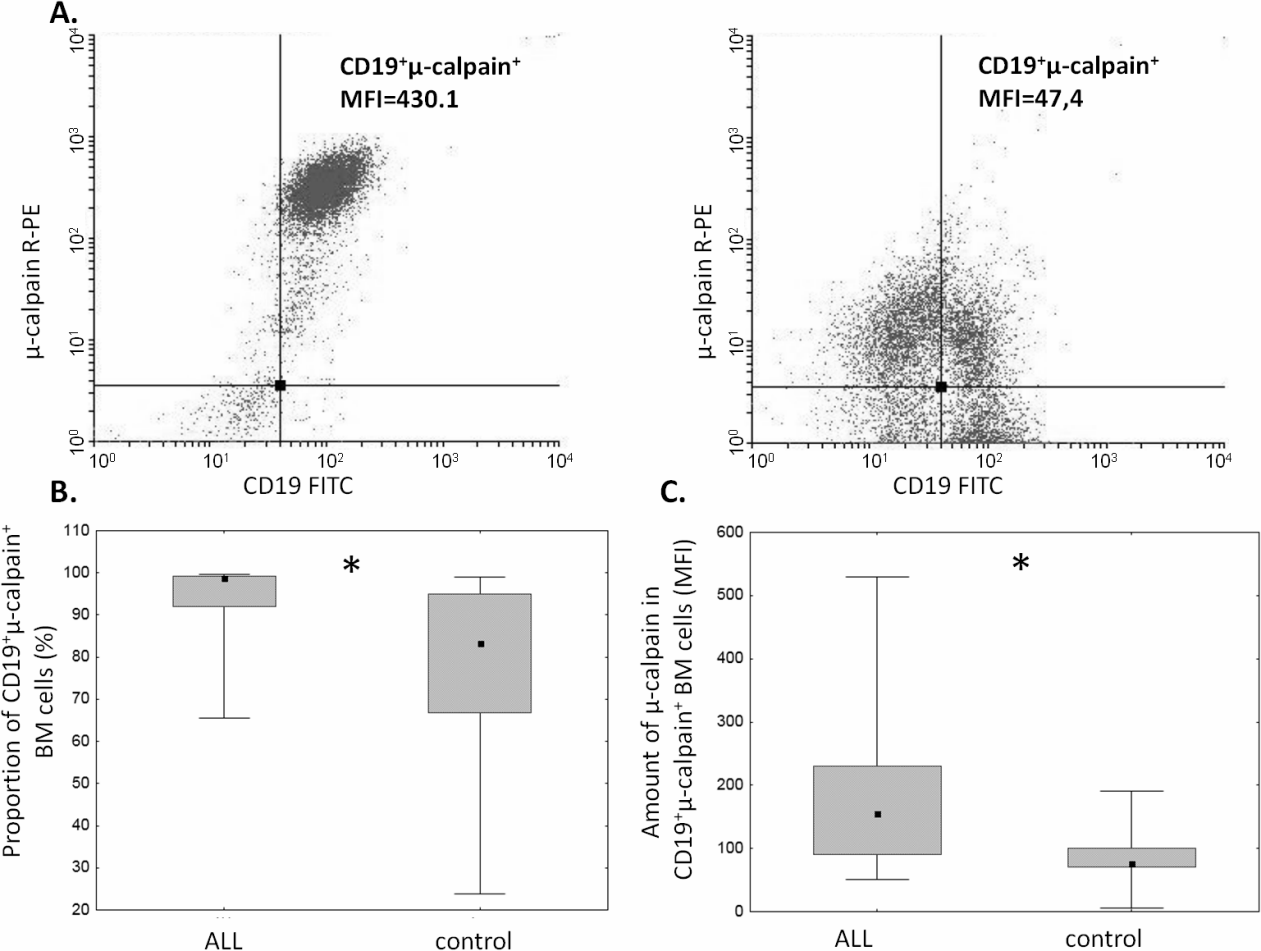
Proportions of μ-calpain-positive cells and relative amounts of μ-calpain are elevated among ALL blasts. A. Representative two-parameter plots (dot plots) resulting from simultaneous staining of BM samples (left panel–ALL, right panel–control) with anti-CD19, anti-CD34 and anti-μ-calpain antibodies. Actual corrected MFI values for calpain signal in CD19^+^ cells are shown. B. Significant difference between the proportion of μ-calpain-positive cells among ALL blasts and nonmalignant BM B cells. Box-and-whisker plots depict the medians, 25^th^ and 75^th^ percentile and range respectively. Asterisk signifies p = 0.02; N(ALL) = 20, N(control) = 9. C. Amount of μ-calpain is significantly higher in ALL blasts than in nonmalignant B lymphocytes. Comparison of relative intensities (MFI) of μ-calpain–bound antibody in CD19^+^ ALL blasts (ALL) and non-malignant B cells (control). Asterisk denotes p = 0.03; N(ALL) = 16, N(control) = 9). For the details see Materials and Methods.

**Fig 2:**
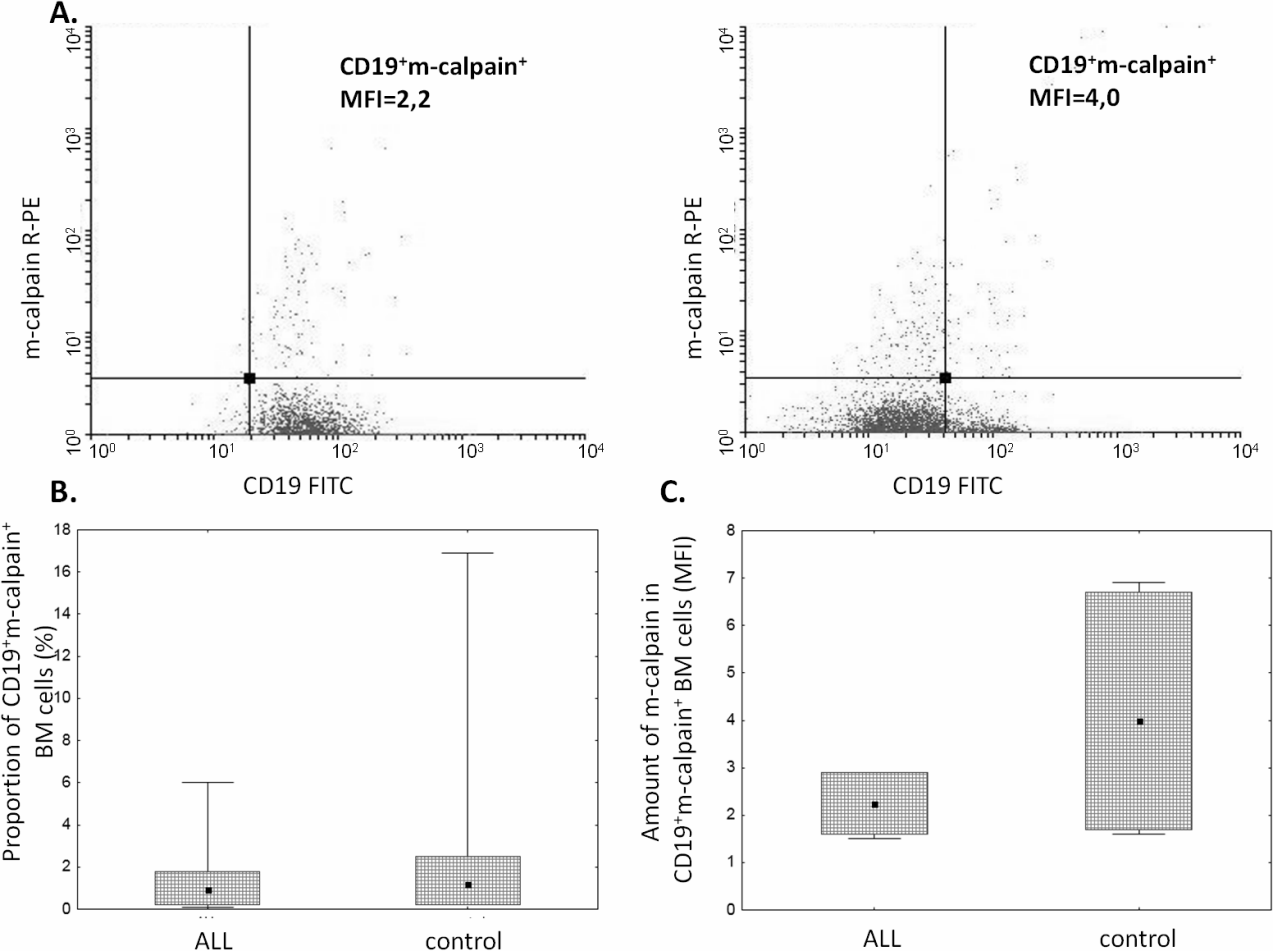
Similar proportions of m-calpain-positive cells and amounts of m-calpain in ALL blasts and control cells. A. Representative two-parameter plots (dot plots) resulting from simultaneous staining of BM samples (left panel–ALL, right panel–control) with anti-CD19 and anti-m-calpain antibodies. Actual corrected MFI values for calpain signal in CD19^+^ cells are shown. Details in Materials and Methods. B, C. No significant difference between the proportion of m-calpain-positive cells among ALL blasts and nonmalignant BM B cells (B) and between amount (MFI) (C). Box-and-whisker plots depict the medians, 25^th^ and 75^th^ percentile and range respectively. N(ALL) = 6, N(control) = 6. For the details see Materials and Methods.

**Fig 3:**
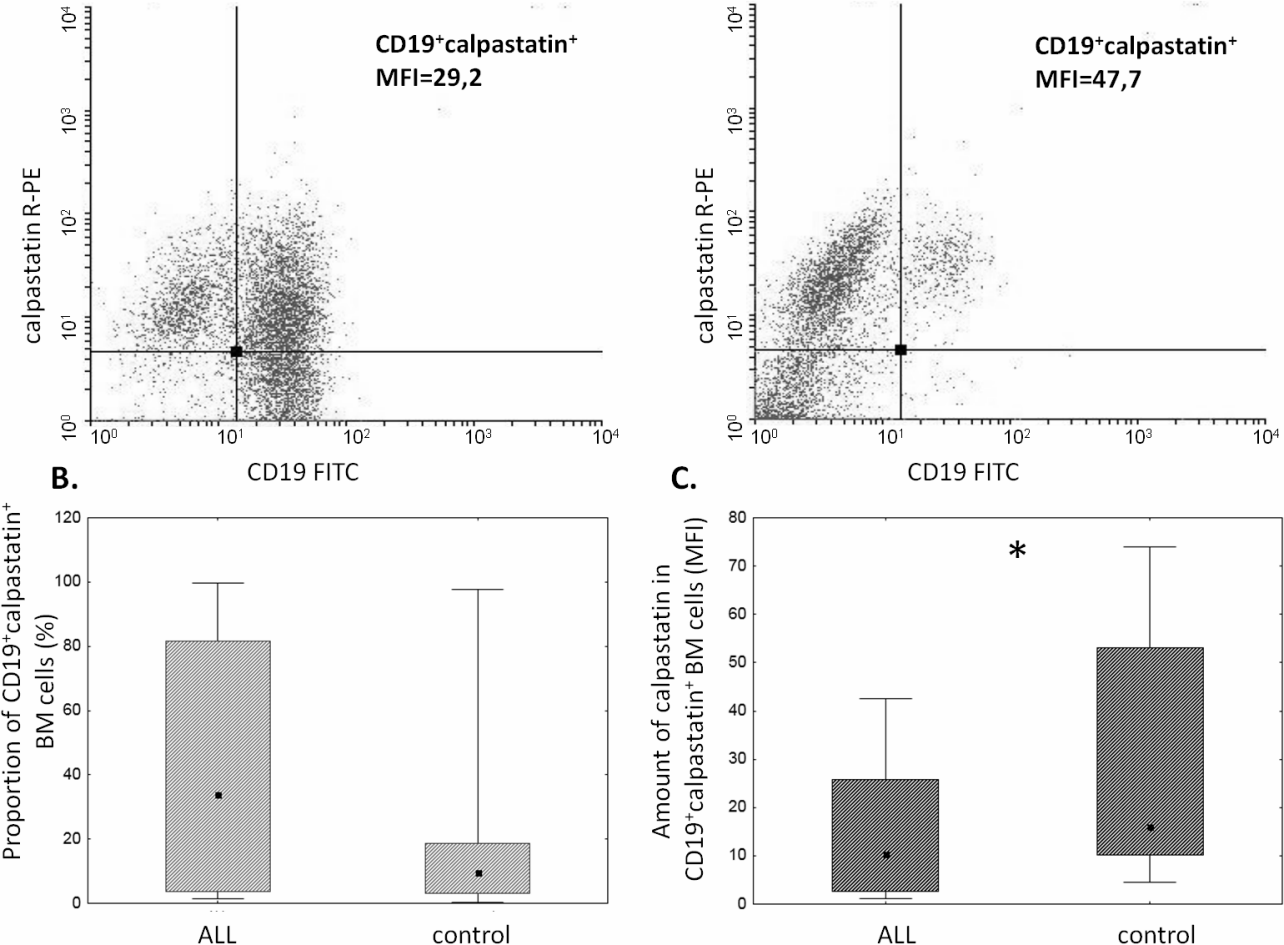
The amounts of calpastatin differ between CD19^+^ ALL blasts and non-malignant B cells. A. Representative two-parameter plots (dot plots) resulting from simultaneous staining of BM samples (left panel–ALL, right panel–control) with anti-CD19 and anti-calpastatin antibodies. Actual corrected MFI values for calpastatin signal in CD19^+^ cells are shown. B. No significant difference between the proportions of calpastatin-positive cells among ALL blasts and nonmalignant BM B cells. C. Significantly lower calpastatin amount (MFI) in the blasts (C). Box-and-whisker plots depict the medians, 25^th^ and 75^th^ percentile and range respectively. N(ALL) = 30, N(control) = 17. For the details see Materials and Methods.

The proportion of μ-calpain^+^ cells was significantly higher among CD19^+^ ALL blasts compared to B lymphocytes in the control group (Fig 1B). In the light of published data, assuming generally that μ-calpain is present in all leucocytes[10,42] the only explanation of these percentages being lower than 100% is that in some B lymphocytes the levels of this enzyme were below the detection threshold of the method used. Also the assessment of μ-calpain amounts measured semi-quantitatively as MFI in ALL blasts and non-malignant CD19^+^ cells showed significantly higher amount of this enzyme in the blasts (Fig 1C). Unlike that of μ-calpain, the percentage of m-calpain^+^ cells as well as the m-calpain MFI did not differ between CD19^+^ ALL blasts and healthy CD19^+^ cells (Fig 2). It is worth noting here that the proportion of m-calpain-expressing cells was extremely low, as was the amount of the enzyme in the cells, regardless their benign or malignant character. Finally, although the cytometrically assessed proportions of calpastatin-positive cells did not differ between the BM CD19^+^ ALL blasts and nonmalignant B cells, the actual levels of calpastatin expressed as corrected MFI were significantly lower in the blasts (Fig 3). As ALL blasts of the patients were confirmed to be CD19^+^CD34^+^, in some experiments the processed BM cells from control individuals were gated for CD34 positivity, then the levels of expression of calpains were additionally assessed in the non-malignant CD19^+^CD34^+^. We did not see any difference between the detected amounts of all three CCS proteins when they were compared in the general BM CD19+ and in the CD19^+^CD34^+^ subpopulation of non-malignant BMs (not shown).

### 2. Comparison of the CCS genes’ expression

**Fig 4:**
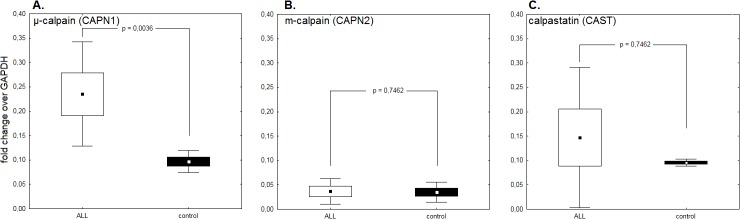
Expression of μ-calpain but not other CCS genes is different in ALL blasts and nonmalignant BM B cells. A. Significantly higher expression of *CAPN1* (μ-calpain) gene in ALL blasts compared to control B cells. B,C. No differences between expression of *CAPN2* (m-calpain) and *CAST* (calpastatin) genes in ALL blasts vs non-malignant B cells. Please mark huge variability of expression of both the *CAPN1* and especially *CAST* genes. CCS gene expression is shown as proportion of the expression of *GAPDH* housekeeping gene considered 1. Box-and-whisker plots depict the means, SEM and SD respectively. P values (Kruskall-Wallis test) are given in the graphs; N(ALL) = 6, N(control) = 6. For the details see Materials and Methods.

The differences in CCS protein amounts shown above prompted our interest in the levels of transcription of respective genes. Using quantitative real time PCR and the ΔΔCt method for results analysis we could demonstrate that the expression of μ-calpain gene (*CAPN1*, Fig 4A) was significantly higher the ALL blasts compared to the B cells from the control group (mean fold change ALL vs control = 2.3), while the expression of both m-calpain (*CAPN2*, Fig 4B) and calpastatin (*CAST*, Fig 4C) genes did not differ between the compared groups (fold changes 0.93 and 0.895 respectively). Interestingly, expression of all three CCS genes in the control cells was very uniform and, in case of *CAPN1* and *CAST*, practically at the same level, while that of *CAPN1* and especially *CAST* gene in the blasts was widely dispersed among the samples.

### 3. Assessment of the endogenous calpain activity in ALL blasts ex vivo

**Fig 5:**
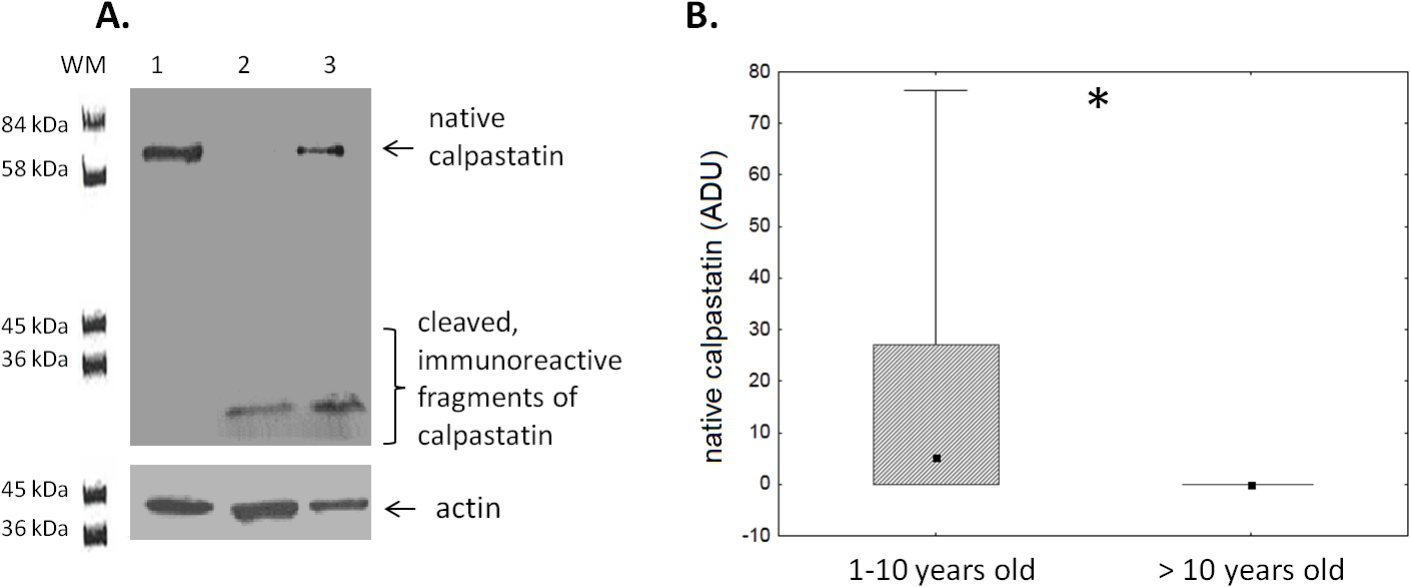
Endogenous calpain activity is present in ALL blasts. **A**. Representative result of western blot determinantion of calpastatin and its immunoreactive fragments resulting from calpain activity in non-malignant BM CD19^+^ cells (lane 1), ALL blasts from a 12-year old patient (lane 2) and blasts from the same patient as in lane 2, but incubated in vitro for 24 hours with 4 μM calpain inhibitor IV (lane 3). Actin was used as a reference protein. For further details see Materials and Methods. B. Endogenous calpain activity in ALL blasts measured by degree of calpastatin degradation (loss of the native form) is significantly higher in the children more than 10 years old. Box-and-whisker plots depict the medians, 25^th^ and 75^th^ percentile and range respectively. Asterisk signifies p = 0.01; N (1–10 years old ALL patients) = 27, N(>10 years old ALL patients) = 10.

Our own method based on the detection of calpain-specific degradation of cellular calpastatin was used; see Materials and Methods for details[35]. All 37 samples of ALL blast lysates tested contained specifically degraded calpastatin indicating endogenous calpain activity, with the majority (27/37) showing variable activities ranging from limited to almost total degradation of available calpastatin (not shown). No calpain activity could be detected in nonmalignant BM B lymphocytes using the same technique. A representative result of one such experiment, where lysates from non-malignant BM B cells, ALL blasts from a 12 year-old patient and the same blasts treated with 4 μM CI IV for 18 hours are tested for calpastatin expression by Western blot is shown in the Fig 5A. Detection of some native calpastatin in the calpain inhibitor-treated blasts clearly shows that calpain is indeed responsible for calpastatin cleavage in the blasts and that the method can be used for the determination of endogenous calpain activity. As patient age is a risk factor, with children older than ten years being at higher risk associated with poorer response after relapse (revieved by Bhojwani and Pui[43]) and decreasing event-free survival (reviewed by Hochberg et al., [44]), we have examined whether this endogenous calpain activity in the ALL blasts is different if our patients were subdivided according to age. The older patients (age>10 years) had on average demonstrated high to very high calpain activity (in no sample from that group had native calpastatin been detected) (Fig 5B).

### 4. Analysis of the in vivo and in vitro relation between ALL blast apoptosis and CCS system

**Fig 6:**
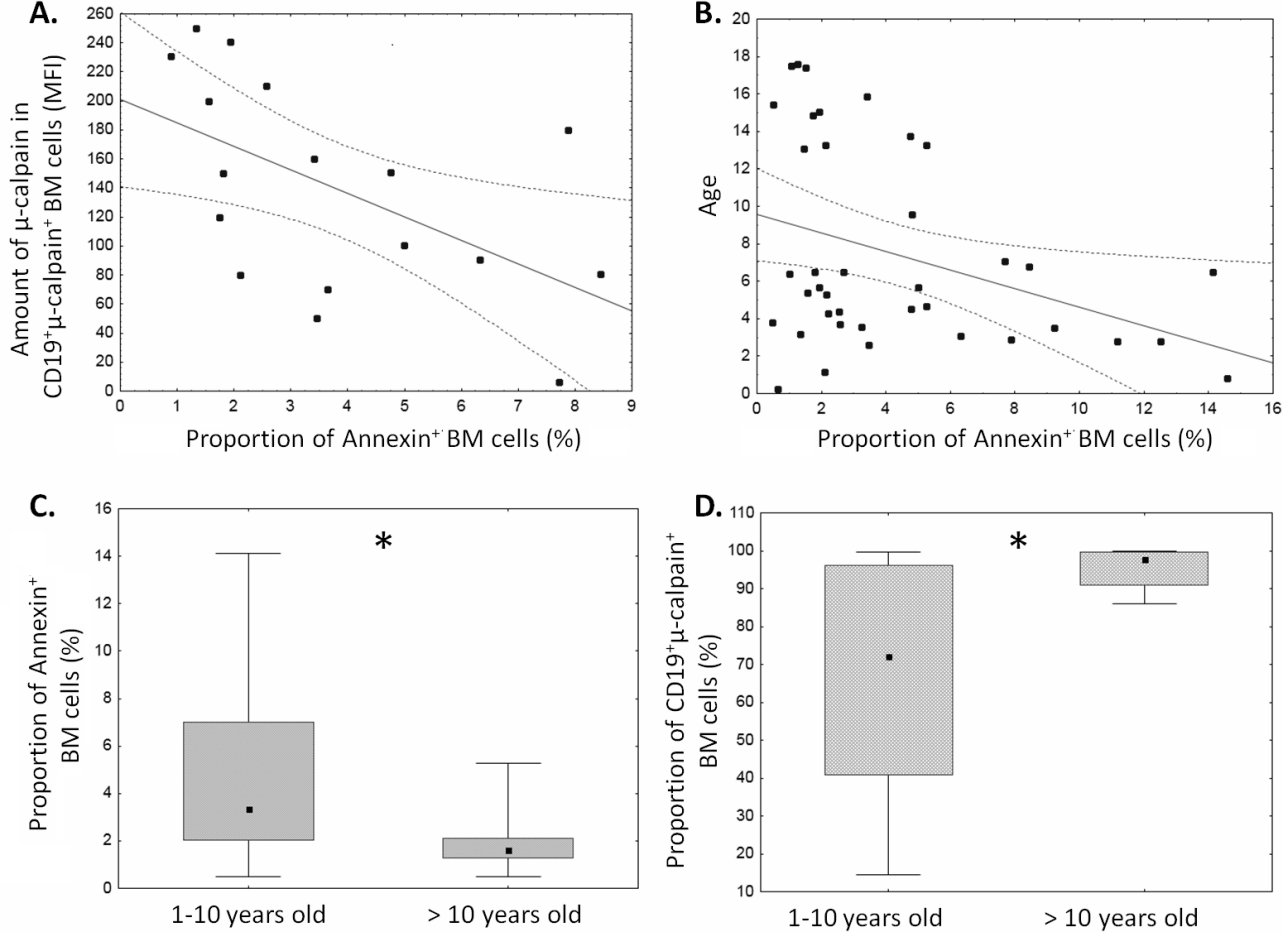
Levels of spontaneous apoptosis of ALL blasts depend on patient age and μ–calpain amount. Apoptosis was determined as the proportion of AnnexinV+ blasts and plotted against the amount of μ–calpain in the blasts (A, N = 17, r = -0.54, p< 0.05) and against patients’ age (B, N = 39, r = -0.31, p< 0.05). See Materials and Methods for details. When the patients were subdivided into below and above 10 years of age subgroups, the latter were characterized by significantly lower apoptosis (C, asterisk signifies p = 0.01) and significantly higher proportion of the μ–calpain positive blasts (D, asterisk signifies p = 0.04). N (1–10 years old ALL patients) = 29, N(>10 years old ALL patients) = 10.

Spontaneous apoptosis of ALL blasts measured after 18 hours in vitro ranged from 1 to 14% (Fig 6). This variation allowed us to seek possible relation between the level of ALL blast apoptosis ex vivo and the amounts of μ-calpain and proportions of calpain-positive blasts. Significant *inverse* relation between the amount of μ-calpain [MFI] and spontaneous apoptosis was noted in ALL blasts (Fig 6A). Similarly significant negative correlation between the percentage of apoptotic cells and the age of the ALL patients was found—the older the ALL patients, the stronger inhibition of apoptosis in ALL blasts (Fig 6B). Also, when patients were subdivided into age groups (below and above 10 years of age), the older subgroup exhibited significantly lower proportion of spontaneously apoptotic ALL blasts simultaneously with significantly higher proportion of μ-calpain-positive blasts (Fig 6C and 6D). Finally, despite relatively short time of follow-up, we had already observed a significantly lower proportion (p = 0.03) of spontaneously apoptotic cells with higher *μ*-calpain amount and activity in our 6 patients who, at the end of follow-up period, were recorded to exhibit adverse events (relapse followed by bone marrow transplant in 3/6 or by death in another 3/6) versus those 33 exhibiting event-free survival.

**Fig 7:**
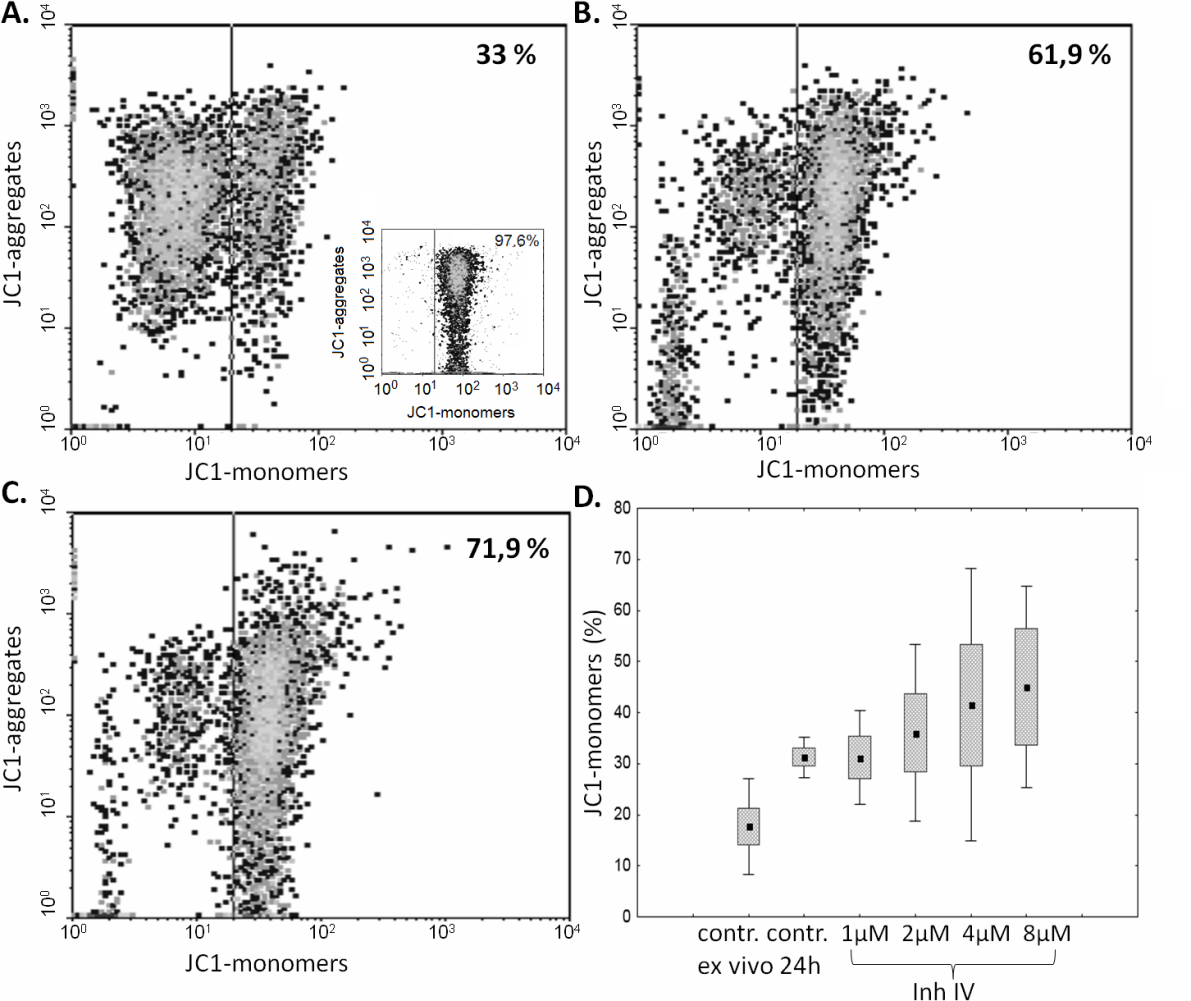
Inhibition of calpain activity in vitro induces ALL blast apoptosis in a dose-dependent manner. Representative cytometric result of the changes in the mitochondrial potential (relative increase of JC1 monomer fluorescence) in ALL blasts incubated over 24 hours without (**A**) and with calpain inhibitor IV at concentrations of 2 and 4 μM (B, C). An insert in A shows the result of a positive control experiment, where BM blasts were treated with 5 μg/ml chelerythrine. D—The effect of calpain inhibitor IV concentration on apoptosis of ALL blasts. Box-and-whisker plots depict the medians, 25^th^ and 75^th^ percentile and range respectively; N = 6. See Materials and Methods for details.

As the ex vivo studies presented above strongly suggested the relation between the amount and activity of μ-calpain and spontaneous apoptosis of the ALL blasts, we decided to check if we can induce blast apoptosis by inhibiting the enzyme activity. A significant, calpain inhibitor dose-dependent increase in the percentage of cells containing monomeric form of JC1, corresponding to a severe mitochondrial depolarization (i.e., increase of early apoptosis), was shown in blasts treated with calpain inhibitor IV for 24 hours. The strongest induction of blast apoptosis have been noticed when the cells were treated with 2–8 μM calpain inhibitor IV, while 1 μM had no effect. (Fig 7). Pretreatment of blasts with 20 μg/mL calpain inhibitor II for 24 hours produced the effect similar to that of 4 μM calpain inhibitor IV, proving that the effect is not related to direct cytotoxicity of the single inhibitor (not shown).

## Discussion

The ubiquitous calpain-calpastatin system (CCS) activities are long recognized and well documented to participate in the cellular proliferation and apoptosis, both in nonmalignant and cancer cells; reviewed in Łopatniuk and Witkowski[21] and Storr et al[45]. We have shown before that excessive amount and activity of μ-calpain is associated with reduced ability of chronic B-cell leukemia (B-CLL) cells to undergo apoptosis[10]. The only other report published so far on the activity of calpains in hematological malignancies is that by Niapour et al, demonstrating that in acute myelogenous leukemia the calpain activity was also greatly elevated and inversely correlated with calpastatin levels; also, the calpain activity correlated with patients response to treatment[30].

Similarly to the B-CLL cells, the ALL blasts are known to escape both spontaneous and induced apoptosis, the feature which may not only quicken the development of the disease, but also lay basis to relatively frequent therapeutic failure, including no or delayed remission, early relapse and death[1,46,47]. We show here that not only there is an increased amount of the μ-calpain in the ALL blasts, but the enzyme is actually (potentially permanently) active (unlike in non-malignant, resting lymphocytes). This in vivo activity leads to degradation of the natural inhibitor of calpains–calpastatin–coexisting with the protease in the cytoplasm, and thus shifts the stoichiometry of the CCS towards an uncontrolled proteolysis of the relevant substrates. At the functional level, we show here that not only the cellular levels of μ-calpain strongly correlate with spontaneous apoptosis of the blasts (Fig 6), but also it is possible to significantly (on average more than twofold) increase the rate of ALL blast apoptosis in vitro by treatment with membrane-penetrating calpain inhibitor (Fig 7).

One of the well known risk factors in childhood ALL is patient age above 10 years, associated with poorer response after relapse (reviewed in[43]); and decreasing event-free survival (reviewed in[44]). Despite a relatively small group of patients under study, we were able to show that both the proportion of ALL blasts with detectable levels of μ-calpain, as well as the endogenous activity of the enzyme was significantly higher in the patients older than 10 years, correlating with lower proportion of the blasts entering spontaneous apoptosis (Fig 6).

Interestingly, BM B cells and ALL blasts seem not to have significant amounts of m-calpain, very unlike normal peripheral blood T and B cells, and even the B-CLL cells[10,48]. At this moment it is difficult to speculate if lack of the m-calpain is of any consequence for the ALL blast biology; thus, further studies would be indicated.

What could be the molecular mechanism of the observed increased amount and endogenous activity of μ-calpain in ALL blasts? Our analysis of transcription levels of all three CCS genes (*CAPN1*, *CAPN2* and *CAST*) had demonstrated that only the activity of *CAPN1* (μ-calpain) gene was significantly, on average more than twice, higher in the blasts than in the control B cells; we have demonstrated earlier that this was the case for B-CLL cells[10]. *CAPN1* transcription levels (Fig 4) paralleled the relative amounts of the μ-calpain protein detected in the ALL blasts and non-leukemic BM B cells respectively (Fig 1). The same was true for two remaining CCS members, m-calpain and calpastatin, where the transcription levels of respective genes did not differ significantly between ALL and control samples. Cytometric analysis of calpastatin levels in the ALL blasts is showing relatively large proportion of cells with no detectable calpastatin, while practically all BM B cells from control subjects had similarly high levels of the protein (Fig 3C and 3D). Our analysis of transcriptional activity of calpastatin (*CAST*) gene in ALL blasts and BM B cells demonstrated great variability in the former, and very tight distribution in the latter, in some ALL cases being close to nul which migt be responsible for lack of calpastatin protein in these cells. One can speculate that decreased transcriptional activity of the *CAST* gene may result in relatively lower amounts of calpastatin (which we had observed at least in some blast samples) and, possibly, slower replacement of the inhibitor being used up by calpain activity (as we demonstrate in the Figs 3 and 5, respectively), thus strongly participating in the shifting of the stoichiometric balance within the CCS in ALL blasts towards relatively uncontrolled proteolysis by “unchecked” calpains. It is also possible that the endogenous calpain activity we observed in the ALL blasts was at least in some cases strong enough to degrade the available calpastatin beyond the possibility of immunological detection by FACS, assisting to lower transcription of its gene. There is an interesting possibility here, related to the fact that *CAST* (calpastatin) gene resides in the 5q15 region of the fifth chromosome, which was reported to be partially or altogether deleted at least in some cases of childhood ALL[49–52]. However, karyotype analysis of our patient samples yielded just one (1/39) case of the deletion in 5^th^ chromosome and a single case of translocation between the 5^th^ and 10^th^ chromosome, which speaks against the involvement of such mutation in the phenomenon observed. On the other hand, reported common childhood ALL karyotype changes (chromosomal mutations) do not involve the chromosome 11, being the site of *CAPN1* gene (reviewed by Lo Nigro[53]), which seems to corroborate with us being unable to demonstrate significant differences in the overall *CAPN1* transcription levels between ALL and non-ALL samples. In fact, we had recorded a translocation involving chromosome 11 –t(4,11)q21,q23 –in a single case among our ALL patients. Of course one cannot exclude the possibility of some other, possibly more common, ALL-associated mutation resulting in the observed downregulation of the *CAST* gene in ALL blasts, perhaps via the change in the pattern of expressed miRNAs. However, at the moment, there are no known/reported miRNAs associated with the expression of large subunit of *CAPN1*. Still, at this time it would be a pure speculation to say what mechanisms (genetic? epigenetic? other) lie behind the increased transcription of *CAPN1* and variability of *CAST* gene transcription observed in the blasts and the issue requires further investigation.

Another precondition for μ-calpain activity is Ca^2+^ concentration in the cytoplasm exceeding the typical resting level of 100 nM and, at least locally, approaching micromolar concentrations. Is such a precondition fulfilled in the ALL? Interestingly, ALL is frequently complicated by hypercalcemia, a condition which is relatively frequent and more typical for older children[54], and sometimes considered a harbinger of the disease[55]. Thus, extracellular levels of Ca^2+^ may be elevated in ALL patients, facilitating its entry into the cytoplasm. On the other hand, there are (unfortunately scarce) reports demonstrating elevated intracellular Ca^2+^ in ALL blasts, for instance in those cases of ALL where BCR-Abl kinase was present and active[56],. Elevation of cytoplasmic Ca^2+^ concentration in the pre-B ALL cells may be induced by CXCL12 chemokine stimulation[57]. CXCL12 levels were reported to be elevated in ALL[58], making feasible such a mechanism of cytoplasmic Ca^2+^ increase. Finally, also the cytokine bFGF is strongly inducing intracellular Ca^2+^ in ALL blasts, for which it is a pro-survival factor[59]. Increase in circulating bFGF in ALL has been reported[60]; we had also observed relatively increased bFGF concentrations in the sera of our ALL patients (Ruckemann-Dziurdzińska et al, in preparation). Summarizing, ALL blasts seem prone to elevated intracellular Ca^2+^ which may be responsible for observed endogenous activation of μ-calpain in these cells.

How is elevated activity of μ-calpain preventing apoptosis of the ALL blasts? One potential target is an improper accumulation of c-myc, normally posing a substrate for calpains, but in ALL blasts demonstrated to be hyperphosphorylated, which might prevent it from being degraded[61]. Recently an interesting model was proposed by Li et al in the context of B-cell lymphoma[62]. There, increased activity of c-myc was shown to reduce the amount of calpastatin (a phenomenon already seen by Niapour et al[63]. Imbalance of calpain/calpastatin stoichiometry would lead to activity of calpain resulting in removal of an (unspecified) caspases-3 activator or direct degradation of the effector caspases. In parallel, calpain activity would increase pro-survival and proliferation signals in the lymphoma cells. We have seen earlier a direct reciprocal relation between high calpain activity and low caspase-3 amount and activity in the B-CLL cells[10]. Our observations of reduced amounts and degradation of calpastatin in ALL blasts seem to corroborate this mechanism.

Concluding, the assessment of the levels and activities of CCS proteins and our successful attempt to modulate their activity leading to the induction of blast apoptosis may help to understand the pathomechanism of ALL better; it may also contribute to the development of new prognostic markers and possibly therapeutic strategies, where μ-calpain, and/or perhaps calpastatin may become potential targets for new (supplementary) anti-ALL therapy. As shown by our data presented here, increased *CAPN1* amount and activity is more likely to occur in the patients older than 10 years, who also do have lower levels of spontaneous blast apoptosis (Fig 6). They would be one of the two primary targets for adjuvant anti-calpain therapy, the other being children with detected 5q chromosome deletion.
