# Correction: Increased μ-Calpain Activity in Blasts of Common B-Precursor Childhood Acute Lymphoblastic Leukemia Correlates with Their Lower Susceptibility to Apoptosis

**DOI:** 10.1371/journal.pone.0139063

**Published:** 2015-09-18

**Authors:** The PLOS ONE Staff

The fourth author’s name is incorrectly spelled in the PDF version of the article. The correct name is: Joanna E. Frąckowiak. The publisher apologizes for the error.
